# Klippel-Feil syndrome accompanied by partial cleft of the cervical spine: a not-so-unusual combination?

**DOI:** 10.1590/0100-3984.2017.0123

**Published:** 2019

**Authors:** Vítor Lopes Galvão Vieira, Debora Bertholdo

**Affiliations:** 1 Hospital de Clínicas da Universidade Federal do Paraná (UFPR), Curitiba, PR, Brazil.

**Figure 1:**
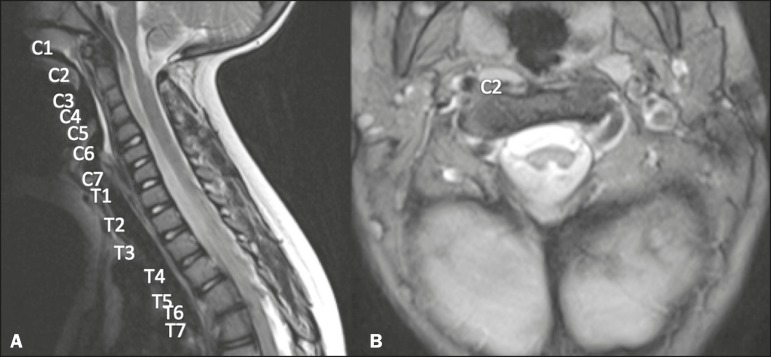
Case 1. **A:** Fusion of cervical vertebrae, extending from C2 to C5. **B:** Posterior division of the spinal cord.

**Figure 2:**
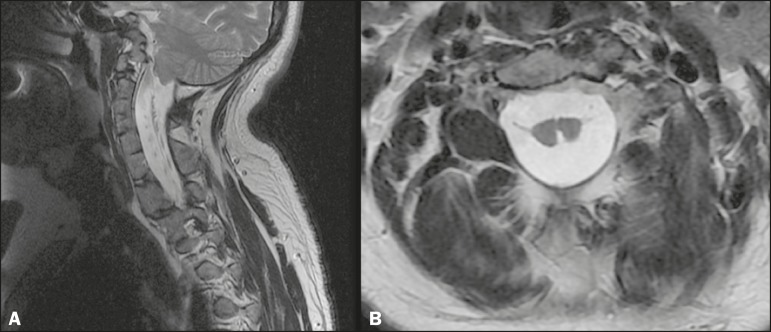
Case 2. **A:** Extensive fusion and deformity of the cervical vertebrae and the first thoracic vertebrae. **B:** Posterior split of the spinal cord, extending from the medulla oblon- A B gata to the first thoracic vertebrae.

Dear Editor,

In this report, we present two cases within the spectrum of Klippel-Feil syndrome (KFS) accompanied by posterior partial split of the spinal cord. The first case involved a woman who underwent magnetic resonance imaging (MRI) of the cervical and thoracic spine to investigate the presence of hemivertebrae and scoliosis. The images showed fusion from C2 to C5, together with posterior spinal cord cerebrospinal fluid cleft (Figure 1). The second case involved an adolescent male hospitalized for cerebrospinal fluid shunt valve replacement and correction of hydrocephalus. MRI revealed extensive fusion and deformity of the vertebrae throughout the cervical segment and superior thoracic segment, accompanied by a long posterior cerebrospinal fluid cleft that extended from the medulla oblongata to the superior thoracic segment of the spinal cord (Figure 2).

KFS is characterized by congenital fusion of two or more vertebral bodies, of unknown cause. Etiological hypotheses include injury occurring at 3-8 weeks of gestation and an association with the genes that control the process of embryonic somite formation^(^1^)^. The classic clinical triad, as described by Maurice Klippel and André Feil, consists of shortening of the neck, low posterior hairline, and limited range of motion of the neck, being present in only 50% of cases^(^2^)^. A wide range of brain and spinal cord malformations have been reported in patients with KFS. Diastematomyelia, one of the most widely reported malformations accompanying KFS, is characterized by complete division of the neural tube and the formation of two hemicords separated by osseocartilaginous septum, typically in the lower thoracic and lumbar regions^(^3^)^. Among the few reported cases of KFS, spinal cord alterations occur in up to 50%, and posterior spinal cord cleft (also known as partial diastematomyelia) is one such alteration^(^4^-^6^)^.

The relationship between vertebral fusion (KFS) and posterior partial spinal cord cleft is poorly documented and does not have a plausible origin like the defects involving the duplication of the notochord, which would explain in a more appropriate way the formation of diastematomyelia and the production of hemivertebrae or the so-called "butterfly" vertebrae^(^1^)^. One of the proposed mechanisms of vertebral fusion is extension of the cartilage formation process to the intervertebral disc material after completion of the primary segmentation (given that there is no change in the number of nerve roots), culminating in the union of the vertebrae^(^1^)^. Spinal cord cleft has an even more obscure origin, and it has been suggested that it is due to focal injury with a superficial tissue repair process, although without repopulation with somite cells^(^1^)^.

In patients with KFS, spinal cord changes are considered to be one of the most important factors in the neurological deterioration process, second only to nerve involvement caused by degenerative spondylosis^(^4^-^6^)^. Because of the small number of cases reported, we raise the question about the actual prevalence of this combination and reiterate the need for active case finding, given that isolated KFS seems to be much more common in clinical practice than is the combination of KFS and spinal cord malformation, especially now that MRI has become much more accessible.
